# From protein interactions to functional annotation: graph alignment in *Herpes*

**DOI:** 10.1186/1752-0509-2-90

**Published:** 2008-10-28

**Authors:** Michal Kolář, Michael Lässig, Johannes Berg

**Affiliations:** 1 Institut für Theoretische Physik, Universität zu Köln, Zülpicher Straße 77, 50937 Köln, Germany; 2 Institute of Molecular Genetics, Academy of Sciences of the Czech Republic, Vídeňská 1083, 14220 Praha, Czech Republic; 3 Kavli Institute for Theoretical Physics, University of California, Santa Barbara, CA 93106-4030 Santa Barbara, USA

## Abstract

**Background:**

Sequence alignment is a prolific basis of functional annotation, but remains a challenging problem in the 'twilight zone' of high sequence divergence or short gene length. Here we demonstrate how information on gene interactions can help to resolve ambiguous sequence alignments. We compare two distant *Herpes *viruses by constructing a *graph alignment*, which is based jointly on the similarity of their protein interaction networks and on sequence similarity. This hybrid method provides functional associations between proteins of the two organisms that cannot be obtained from sequence or interaction data alone.

**Results:**

We find proteins where interaction similarity and sequence similarity are individually weak, but together provide significant evidence of orthology. There are also proteins with high interaction similarity but without any detectable sequence similarity, providing evidence of functional association beyond sequence homology. The functional predictions derived from our alignment are consistent with genomic position and gene expression data.

**Conclusion:**

Our approach shows that evolutionary conservation is a powerful filter to make protein interaction data informative about functional similarities between the interacting proteins, and it establishes graph alignment as a powerful tool for the comparative analysis of data from highly diverged species.

## Background

With the advent of genome-wide functional data, cross-species comparisons are no longer limited to sequence information. A classic extension of sequence alignment is structural alignment, which has been used to compare evolutionary distant RNAs [1] and proteins conserved in structure rather than sequence [2,3]. Here we use protein interactions as evolutionary information beyond sequence [4].

We perform a cross-species analysis of two herpesviruses, the *varicella-zoster virus *(VZV), causing chicken pox and shingles, and the *Kaposi's sarcoma-associated herpesvirus *(KSHV), responsible for cancer of the connective tissue. The two viruses have diverged approximately 200 million years ago. Their sequence dynamics is characterised by a high rate of point mutations (at least an order of magnitude faster than their host populations [5]) and a high rate of gain and loss of genes (an order of magnitude higher than the mutation rates of prokaryotes [6]). As a result, homologous proteins have an amino acid sequence identity of only about 20%. Moreover, many open reading frames are only about 60 amino acids long. Thus, the sequence similarity between the two species is in the 'twilight zone' of detection by alignment, *i.e*., orthologous open reading frames have alignment scores just marginally above the background of unrelated sequences.

To improve the cross-species comparison, we jointly use the similarity of coding sequences and of protein interactions. Our hybrid comparison method called *graph alignment *establishes a mapping between genes of two species [7] using a probabilistic scoring system based on evolutionary rates of sequences and interaction networks. Several recent studies have used orthologs identified by sequence similarity to compare networks, for instance to identify ancestral networks [8], network parts enriched in conserved links [9-11] or to decide between paralogous genes, see [12]. Here this approach is turned on its head: we use network information to identify evolutionary and functional relationships in cases where there is no detectable sequence similarity. Related approaches appeared in [13-16], reviewed in [17,18].

However, these approaches use ad-hoc scoring parameters, or parameters derived from a database of known orthologous genes [16] to determine the alignment. Our method uses an evolutionary model to infer all necessary parameters from the data set itself. In ref. [7] we have applied this method to co-expression networks, which are fully connected. Here we explore the complementary regime of sparsely connected networks with noisy link and node similarity data, where graph alignment is used to resolve the twilight regime of evolutionary correlations. In this regime, statistically significant alignments have to be distinguished from a *low-fidelity regime *of spurious graph alignments. Understanding the statistics of graph alignment in both regimes turns out to be important for validation of the results in the twilight regime.

**Figure 1:**
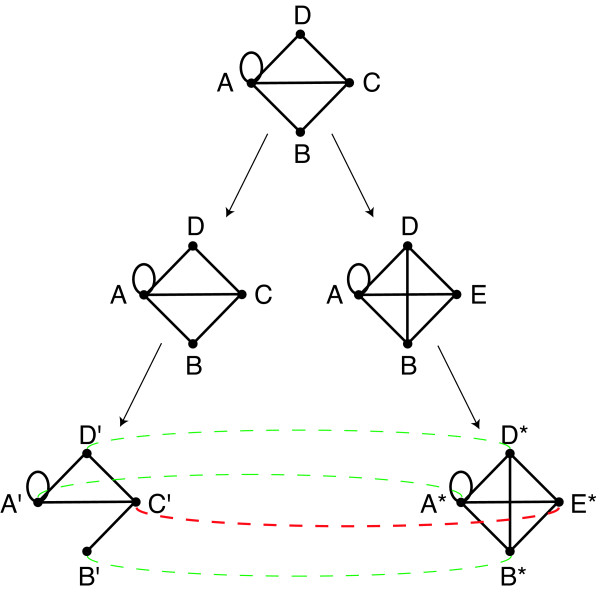
**Detecting functional relationships by graph alignment**. In this example, the gene labelled *C *is replaced in one lineage with its functional equivalent *E*, which has the same interaction partners in the network. While some genes can still be correctly mapped across species using sequence information (green lines), the full evolutionary history and the mapping *C' *- E* are accessible from cross-species analysis only by taking into account the interaction networks.

Our cross-species comparison is grounded on a two-level evolutionary picture for protein coding sequence including (i) the specific sequence parts responsible for protein-protein interactions and (ii) the background coding sequence, most of which is unrelated to these interactions. The relevant processes include divergent sequence evolution, gain and loss of interactions, duplication of genes and the corresponding interactions, and gain and loss of genes. Functional relationships may stem from common ancestry and thus be detectable by sequence *homology*, but they may also arise by convergent evolution, this *analogy *displayed by similar interactions without sequence similarity. An example is given in Figure 1, where one gene has functionally replaced another gene by acquiring its interactions, a process called non-orthologous gene displacement [19]. Similarly, an orthologous gene pair may diverge in sequence beyond detectability, but conserved interaction patterns remain detectable due to functional constraints. Such functional or evolutionary relationships are to be deduced from the network of interactions between genes.

The essence of our graph alignment approach is as follows: Experimental data on interacting proteins defines a protein interaction network consisting of nodes (proteins) and links (protein interactions). At this point, nodes can simply be labelled by a protein name, or ORF identifier, without recourse to sequence information. The local link similarity between pairs of aligned nodes defines the link score of the alignment. Aligned nodes can either both interact (resulting in a positive link score, *e.g*. nodes *A'*, *D' *and A*, D* in Fig. 1), both not interact (resulting in a small positive link score), or interact in one species and not in the other (resulting in a negative link score). The sequence similarity between aligned nodes defines their *node score*. The total score is the sum of link and node terms, with scoring parameters depending on the evolutionary distance between the species compared. Finding high-scoring graph alignments is an algorithmically hard problem, and we use the algorithm introduced in [7] to perform the search. However, since many proteins have no clear sequence ortholog *and *few interaction partners, it turns out that high-scoring alignments are not guaranteed to be statistically or biologically significant. There exists a regime of spurious alignments consisting of islands of locally matching topology which do not respect sequence similarity: the low-fidelity regime discussed further in the methods section. It turns out that optimal alignments are produced using scoring parameters in the high-fidelity regime close to the transition to the low-fidelity regime.

For the graph alignment between the VZV and KSHV viruses studied here, both the interaction networks and the gene sequences are crucial to determine functional or evolutionary relationships, while each part of the data by itself is less significant. In particular, we find protein pairs with low sequence similarity for which the interaction similarity strengthens the statistical inference of homology, as well as protein pairs without sequence similarity, which are aligned based on their interactions alone. We use this alignment to make functional predictions, which turn out to be consistent with published gene expression data, as well as gene position and molecular weight. Given a validated alignment, we can quantify the evolution of protein interactions. We find that interactions between functionally related proteins are more conserved than other interactions.

## Results and discussion

### Optimal graph alignment between VZV and KSHV

**Figure 2:**
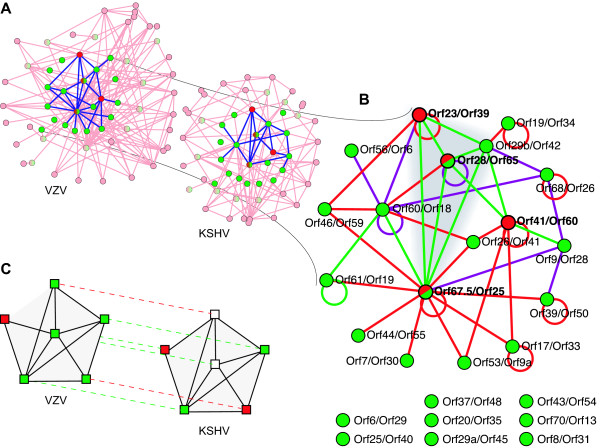
**Alignment of the protein interaction networks of herpesviruses VZV and KSHV**. a) The alignment maps the nodes from the highlighted sub-networks of the PINs. Nodes are colour coded according to sequence similarity, measured by the sequence alignment score *θ *[see Additional file 1]. Green nodes have high sequence similarity with *θ *> 0, red nodes have no sequence similarity detected, red/green nodes have low similarity with *θ *≤ 0. The ORFs that do not belong to the network alignment are shown in pale colours. Protein interactions are represented by links between nodes, interactions between ORFs in the alignment are shown in blue. Supplementary animation [Additional file 2] puts the aligned network further into the context of the PINs. b) The optimal alignment is shown with nodes representing aligned pairs of ORFs. Green links indicate interactions which have been detected in both KSHV and VZV. Interactions which have only been detected in KSHV or VZV are shown in magenta or red, respectively. The cluster of matching interactions linking nodes KSHV ORF23/VZV ORF39, 29b/42, 28/65, and 67.5/25 is highlighted. c) From the alignment to functional annotation: We show the alignment of the VZV ORF65 with KSHV ORF28 (central nodes) and the context in the protein interaction graphs. The aligned partners are connected with dashed lines, the green lines connect ORFs with significant sequence similarity and the red lines connect ORFs that are aligned solely due to similarity of their interactions. An ORF belongs either among structural ORFs (green squares) or information-processing ORFs (red squares), or its function is unknown (white squares). According to the alignment of KSHV ORF28 to VZV ORF65 the KSHV ORF28 is predicted to belong among structural genes. The fact that all but one of its conserved interacting partners have the same functional annotation further supports this prediction (guilt by conserved association).

The protein interaction network of the herpesvirus VZV consists of 76 open reading frames (ORFs) and 173 protein-protein interactions (of these ORFs, 19 have no detected interactions and are disregarded from the subsequent analysis). The protein interaction network of KSHV consists of 84 ORFs and 123 interactions (34 ORFs have no detected interactions), [4], see Figure 2a. Thirty-four ORFs in VZV have reciprocally best matching sequence homologs with reading frames in KSHV. Between pairs of ORFs with such homologous partners, there are 44 interactions in VZV and 25 interactions in KSHV. Of these interactions, 8 occur in both species, that is the overlap between interaction networks is about 13% when the alignment is given by sequence homology. The optimal alignment of the two networks is shown in Figure 2b. The list of aligned ORFs and details on the scoring are given in the supplementary text [see Additional file 1]. The alignment consists of 26 pairs of aligned ORFs, spanning one third of the protein interaction networks of VZV and KSHV. The alignment contains 44 interactions, 10 of which are self-interactions. Of the 34 interactions between distinct ORFs, 11 are matching interactions occurring in both protein interaction networks, only one of the 10 self-interactions matches. Of the 26 pairs of aligned ORFs, 24 pairs have detectable sequence similarity. The remaining 2 aligned pairs involve ORFs which have no detectable sequence similarity with each other or any other ORF. The mean connectivity of the aligned part of the protein interaction network is 3.0 interactions per ORF, compared with a mean connectivity of 2.4 of VZV and 1.5 of KSHV.

**Figure 3:**
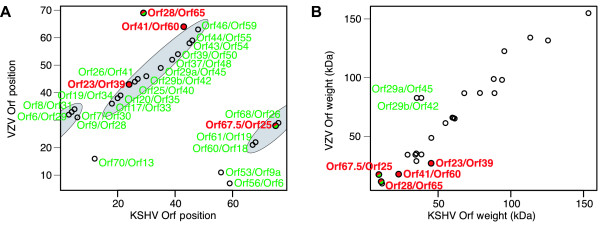
**Corroborating evidence for the network alignment from gene position and molecular weight**. a) The gene rank of reading frames of VZV is plotted against the rank in KSHV of their alignment partner. The points fall into two diagonal bands indicating the conservation of gene order between the two viruses. The ORF pairs aligned solely on the basis of matching interactions fall within those bands. The only significant deviation from those bands, the pair KSHV ORF28/VZV ORF65, has related sequences, see text. b) The molecular weights of aligned pairs of reading frames show a strong correlation (Pearson's correlation coefficient *r *= 0.94). The two exceptions again are aligned because they have related sequences (top left, indicated in green). The aligned ORFs with little or no sequence similarity (red circles, see text) show highly correlated molecular weights.

The quality of the alignment we have obtained can be tested by comparing the genomic positions of the aligned ORFs. We count the ranks of ORFs from the initial terminal repeats of the two genomes (left TR of KSHV, TRL of VZV). In Figure 3a the ranks of reading frames in VZV are plotted against the ranks of their alignment partners in KSHV. Aligned ORFs without any sequence similarity fit very well into the sequence of ORFs in their respective genomes. The molecular weights of the aligned nodes are highly correlated, see Figure 3b. In addition, we find that interactions among the aligned ORFs are more likely to be conserved across several other herpes species, including *herpes simplex virus *(HHV-1) and *murine cytomegalovirus *(mCMV). The mutual information on the interactions in different species within the alignment is 6.6-times higher than for the interactions among ORFs outside of the alignment [see Additional file 1 for details].

In some cases, sequence similar pairs of ORFs are not aligned because of mismatched interactions. As an extreme case an ORF may have several interactions in one species, but none in the other, indicating most likely an unsuccessful yeast-two-hybrid assay (Y2H) experiment. Examples are KSHV ORF64/VZV ORF22, 22/37, 42/53, 36/47, and 33/44.

### Functional relationships detected by interaction similarity

Some ORFs are aligned due to their matching interactions, either with low or with no detectable sequence similarity. We discuss these cases separately.

#### KSHV ORF67.5/VZV ORF25

These ORFs have a sequence identity of only 18% over 76 aa (see Methods for details). They are listed as homologs in the VIDA3 database [20], and both of them are thought to be homologs of the HHV-1 protein U_L_33 [21]. The alignment of these ORFs largely results from 4 matching links out of 5 in KSHV and 12 in VZV (p-value of 4 × 10^-3^, [see Additional file 1]) with a local link score *S*_*L *_= 4.57 versus node score *S*_*N *_= 4.20. Our alignment thus confirms the homology.

#### KSHV ORF28/VZV ORF65

These ORFs have a sequence identity of only 11% over 102 aa. They are not listed as sequence homologs in databases VOCS [22], VIDA3 [20] and NCBI [23]. However, the sequence alignment extends over their complete length, with no gaps. Again, the alignment of these nodes results from 4 matching links out of 4 in KSHV and out of 5 in VZV (p-value of 10^-3^) with a local link score *S*_*L *_= 6.30 versus node score *S*_*N *_= 3.50. Functional annotation is available only for VZV ORF65; it belongs to the membrane/glycoprotein class, most likely it is a type-II membrane protein [24]. The alignment of KSHV ORF28 with VZV ORF65 leads us to predict that KSHV ORF28 also codes for a membrane glycoprotein, see Figure 2c for illustration.

Several experimental studies support this prediction. Gene expression studies show that ORF28 is co-expressed with tertiary lytic ORFs and hence probably falls in the classes of structural or host-virus-interaction genes [25,26]. The expression of ORF28 is affected by blocking DNA replication [27] showing ORF28 is a secondary or tertiary gene. Furthermore, ORF28 has been detected in the virion by mass spectroscopy, leading to a tentative functional classification as a glycoprotein-envelope protein [28]. Finally, ORF28 is a positional homolog of the *Epstein-Barr virus *ORF BDLF3, which is known to encode glycoprotein gp150.

#### KSHV ORF23/VZV ORF39

These ORFs have no significant sequence similarity: although the alignment obtained with *clustalW *[29] has a sequence identity of 18% over 240 aa, it is statistically insignificant; a randomised test yields a p-value of 0.43. A systematic analysis involving a wide range of different scoring parameters does not yield a statistically significant sequence alignment either [see Additional file 1]. The reading frames KSHV ORF23 and VZV ORF39 are aligned purely due to 3 matching interactions out of 4 of KSHV and 4 of VZV (p-value 2 × 10^-2^). The local link score equals 4.47 versus a node score of *-*0.49. Functional classification is available only for VZV ORF39 as a membrane/glycoprotein [20]. The alignment thus leads us to predict that KSHV ORF23 also codes for a membrane glycoprotein.

This prediction is supported by several experimental studies. Again ORF23 is co-expressed with tertiary lytic ORFs [25] and is sensitive to blocked DNA replication [27], so it is a late gene. The expression patterns of ORF23 are similar to those of structural and packaging genes.

#### KSHV ORF41/VZV ORF60

These ORFs have 3 matching interactions out of 3 in KSHV and 6 in VZV (*p *= 2 × 10^-2^), but no significant sequence similarity (The *clustalW *sequence alignment has identity of 12% over 160 aa with p-value 0.94). They are aligned with a local link score of 4.39 versus a node score of -0.49. Both ORFs are functionally annotated. KSHV ORF41 codes for a helicase/primase associated factor [30] and is not affected by blocking DNA replication [27]. On the other hand, VZV ORF60 codes for the glycoprotein L [20,31]. It may be that either of them has a so-far unknown function, leading to the matching protein interactions. This idea finds support in [25], where the expression maximum of ORF41 was found to come after the secondary lytic phase. This is surprising because the transcript is needed already during the secondary lytic phase (DNA replication). No other DNA-replicating gene controlled by a different operon to KSHV ORF41 has an expression dynamics with this property. Such a delay of the maximum of expression may have two reasons: either the transcription of the ORF41 is not controlled after its role is finished, or ORF41 indeed has a hitherto uncharacterised function in the tertiary lytic phase, possibly a structural one.

We also note that ORF41 is specific to the class of *γ*-herpesviruses, of which KSHV is a member. Analogously, ORF60 is *a*-herpesvirus specific. It is possible that the homolog of ORF41 in VZV and the homolog of ORF60 in KSHV were lost as a result of either of these proteins acquiring a new function. This would be an example of non-orthologous gene displacement [19].

### Interaction clusters

The alignment shown in the Figure 2 contains a cluster of proteins all interacting with each other. This cluster comprises the aligned pairs KSHV ORF23/VZV ORF39, 28/65, 29b/42, and 67.5/25 connected by matching links only. The p-value for such a fully connected cluster (a clique) to emerge at random is approximately 5 × 10^-11^. The pair KSHV ORF41/VZV ORF60 discussed above is connected to this cluster by two matching links, forming an almost fully connected cluster of 5 ORFs pairs with 8 of 10 possible links present and matching. Surprisingly, while all the other ORFs in the cluster code for structural proteins (virion assembly and structure proteins), ORF41 of KSHV is annotated as a helicase/primase associated factor, and hence codes a protein involved in DNA replication. The association with structure-related genes may be interpreted as a further evidence towards another function of ORF41 as a structural protein.

This cluster of interacting proteins is also found in a third species, the Epstein-Barr virus EBV, which is of the same viral family as KSHV. Three of the four ORFs of the cluster in KSHV have sequence homologs in EBV, namely ORF23, ORF67.5, ORF29b. All of the corresponding ORFs in EBV are found to interact with each other (Peter Uetz, private communication).

The individual species KSHV and VZV contain further clusters, but these are not conserved across species. For instance, the cluster comprising ORFs 28, 29b, 41 and K10 in KSHV contains genes coding for predicted virion proteins, virion assembly and host-virus interaction proteins. ORFs 25, 19, 27, and 38 forming a fully connected cluster in VZV code for proteins involved in virion assembly, nucleotide repair, metabolism, and host-virus interaction.

### Interaction conservation and protein function

Protein interactions which are conserved across species shed further light on the functional relationship of the interaction partners. We compare the functions of interacting proteins (i) when the interaction is conserved between KSHV and VZV, and (ii) regardless of conservation.

Each annotated protein can be assigned to one of two functional classes: it is either a 'structural protein' (its functional annotation is one of capsid/core protein, membrane/glycoprotein, virion protein, virion assembly), or an 'information-processing' protein (DNA replication, gene expression regulation, nucleotide repair/metabolism, host-virus interaction). We take the functions of two proteins to be similar if both their functional annotations fall into the same class. Based on this classification, we measure the correlation between functional annotations of interacting proteins by mutual information. For conserved interactions, this is nearly 20-times higher than for the set of all interactions (0.107 bits vs. 0.006 bits). Hence, conserved interactions are more likely to connect functionally similar proteins. Conversely, functionally similar proteins have more conserved interactions than functionally unrelated genes. The mutual information between interactions in the two species is nearly ten times higher for pairs of functionally similar proteins than for pairs of functionally different proteins (0.071 bits vs. 0.007 bits).

## Conclusion

### Graph alignment results from sequence and interaction similarity

Protein interactions are encoded in mutually matching binding domains. The evolutionary dynamics of these domains is governed by different evolutionary constraints and hence, by different tempi than the overall coding sequence. Moreover, the sequence of a domain may evolve considerably while its interaction is conserved. Therefore, we treat the experimental interaction data as evolutionary information independent of sequence data. Our alignment of herpesviruses VZV and KSHV yields a cross-species mapping between ORFs based jointly on the correlation between amino acid sequences and on the correlation between their protein interactions. The latter correlation depends both on the evolutionary divergence of the interaction networks, and on experimental noise. This approach is distinct from searching for the overrepresentation of matching interactions among sequence homologs [9-11]. It allows the identification of homology in cases where sequence similarity between two ORFs has decayed to statistically insignificant levels. Resolving the 'twilight zone' of sequence similarity by additional information on protein interactions is particularly relevant for the case of short genes (such as in the present application), or high levels of domain shuffling. Our method also allows to detect functional analogs, *i.e*., proteins with similar interactions but without common ancestry. The resulting alignment is corroborated by genomic position and by molecular weight of aligned ORFs.

### Functional predictions from interaction similarity

We find several cases of ORFs with no detectable sequence similarity which are aligned with each other solely on the basis of matching interactions. There are different possible mechanisms generating this situation; (i) a pair of orthologous genes loses their sequence similarity below the threshold of detectability, (ii) convergent evolution, and (iii) a gene functionally substitutes for another gene. The original gene may then be excised from the genome without phenotypic effect. This process has been termed non-orthologous gene displacement [19]. In all three cases, sequence information is insufficient for functional prediction. Based on the alignment due to matching interactions and on the annotation of one of the alignment partners, we predict the function of several ORFs. These predictions are supported by gene expression experiments and by the genomic position of the ORFs.

### Functional cluster as conserved subgraph

The optimal alignment (Figure 2) contains a cluster of 4 ORFs whose products all interact with each other in both viruses. All members of this cluster belong to a single functional class; they are involved in virion formation and structure and code for tertiary lytic transcripts.

There are other fully connected clusters both in VZV and KSHV, but none of them occur in *both *viruses. These clusters contain proteins in different functional classes; one cluster in VZV contains proteins involved in virion assembly, nucleotide repair, metabolism, and host-virus interaction.

### Guilt by conserved association, evolutionary constraints on network links

The guilt-by-association scheme of assigning like functions to interacting proteins [32] would fail in these cases of non-conserved clusters. However, we can refine this principle to guilt by conserved association, assigning similar functions only to proteins with an interaction in *both *species, which correctly describes the functional correlations in the above clusters. Indeed, while the functional classes of interacting proteins in a single species are only very weakly correlated, pairs of proteins with conserved interactions are more likely to share the same function.

The guilt-by-conserved-association principle might be more than a statistical filter for false positive interactions by cross-species comparison. Interactions between proteins of the same functional class are more likely to be conserved across species than interactions between proteins of different functions, which may indicate a lower rate of evolution of interactions related to function. This, in turn, is consistent with natural selection imposing a specific constraint acting jointly on protein interactions that contribute to a cellular pathway. With data on further species, phylogenetic analysis will shed light on the evolutionary forces at the level of the protein interaction networks, particularly if adaptive events can be traced in the data [33].

## Methods

### Scoring sequence alignments

To account the uneven level of sequence divergence along the herpesviral genome, we optimise scoring parameters of the Needleman-Wunsch algorithm individually for each pair of ORFs. We then normalise the scores in the way that allows comparison of scores obtained with various scoring parameters following [34], [see Additional file 1 for details]. The scores are directly comparable to *i.e*. ClustalW scores.

### Scoring graph alignments

Consider a set of genes (or open reading frames) as nodes of a network, with pair-wise interactions between the corresponding proteins represented as Boolean network links. Given two such networks in related species, we construct a graph alignment, *i.e*., a mapping *p *of nodes of one network to nodes of the other network. This alignment is scored by *interaction similarity *and *sequence similarity *as follows: (i) Aligned node pairs (*i, j *= *π*(*i*)) and (*i', j' *= *π*(*i'*)) contribute a positive *link score *if a link is present both between the pair (*i, i'*) in one network and (*j, j'*) in the other (matching links, such as *D' *- *C' *and D* - E* in the example of Figure 1). A negative contribution results if a link is present in one network, but not in the other (mismatched links, such as *D' *- *B' *and D* - B* in Figure 1). The link score accounts for evolutionary divergence of the interaction networks, as well as for experimental errors in the network data. (ii) An aligned node pair (*i, j *= *π*(*i*)) contributes a *node score *depending on the sequence similarity *θ*_*ij*_, rewarding similarity between aligned pairs and penalising similarity between pairs not respected by the graph alignment.

The total graph alignment score is the sum of independent contributions from sequence similarity and from link similarity. Hence, any high-scoring alignment will contain node pairs aligned primarily due to similarity of their interactions or of their sequences, or of both. Of course, the outcome of the alignment depends crucially on the relative weight of node score and link score. We determine optimal scoring functions self-consistently from the data within a Bayesian framework [see Additional file 1].

### Computation of p-values

We consider pairs of independently generated random networks, and compare them to the alignments found in empirical data. The probability of finding in random networks two nodes with the same or higher interaction overlap as a given alignment is estimated, and serves as a p-value for the corresponding alignment [See Additional file 1 for details.]

### Graph alignment algorithm

We use an iterative algorithm as described in [7] to find the high-scoring graph alignments. This algorithm is based on a mapping to the quadratic assignment problem. At each step, the highest scoring alignment is identified individually for each node, while keeping the rest of the alignment fixed. A certain amount of noise is used to help the alignment to escape from local score maxima, a procedure called simulated annealing [35]. This noise amplitude is gradually decreased to zero, starting from some initial value *T *and an initial alignment of reciprocal best sequence matches. An R-package implementing the graph alignment is available from the bioconductor website [36].

### Alignment regimes and parameter selection

We have performed extensive tests, both on artificially generated networks and on the experimental PIN data, to find the optimal scoring parameters. For network pairs with low link similarity, we have found two different alignment regimes depending on the initial noise level *T*. In the *high-fidelity *regime for values of *T *well below a threshold value *T*_*D*_, the alignment consists mainly of the nodes with sequence similarity, but does not extend much beyond. In the *low-fidelity *regime for *T *above *T*_*D*_, high-scoring alignments contain many link matches (even more than in the biologically correct alignment), but different runs have little overlap and most nodes (even with sequence similarity) are misaligned.

Optimal detection of similarity occurs in the high-fidelity regime for values of *T *just below *T*_*D*_. In this region, the alignment is still guided by sequence similarity, yet extends as much as possible into the set of nodes without sequence similarity.

The occurrence of high-scoring alignments of low significance can be understood intuitively from the special case of two uncorrelated graphs with a narrow range of connectivities. Aligning a pair of randomly chosen nodes with each other, their neighbours, and their next neighbours, etc., will lead to a high link score (possibly offset to some extent by a low node score). There are many such alignments with a high score, yet low statistical significance. These spurious alignments occur for sparse networks at sufficiently low fractions of link matches and low numbers of nodes with sequence similarity. They are comparable to the score islands known in local sequence alignment [37,34,39]. However, unlike sequences with their one-dimensional structure, locally tree-like graphs can generate an exponentially large number of such score islands.

### Reproducibility and robustness

To ensure reproducibility of our results the alignment procedure is repeated several times over in order to record how often a given pair of nodes is aligned. The results are shown in supplementary Fig. 2 [see Additional file 1]. As a conservative pruning procedure, we only consider aligned node pairs which appear in more than half the runs (for comparison, under random matching a given alignment partner appears with probability 1/*N *~ 0.03). The optimal scoring parameters turn out not to change between alignment runs.

## Authors' contributions

All authors contributed equally to the work. All authors read and approved the final manuscript.

## Data deposition

The protein interactions for KSHV strain BC-1 and VZV Oka-parental were taken from the yeast two-hybrid screens (Y2H) of the Peter Uetz lab [4]. The sequences of the two herpesviruses were downloaded from the VOCs database [22] and the NCBI database [23,40,41].

**Accession numbers: Genomes**: KSHV: *Human herpesvirus 8 *strain *cell line BC-1 *(VOCs genome ID 890); VZV: *Human herpesvirus 3 *strain *Oka parental *(VOCs genome ID 921). **KSHV ORFs**: ORF 67.5: provided by Peter Uetz, sequence follows: "MEYASDQLLP RDMQILFPTI YCRLNAINYC QYLKTFLVQR AQPAACDHTL VLESKVDTVR QVLRKIVSTD AVFSEARARP"; ORF 28 [GenBank: NP 572080.1]; ORF 23: [GenBank: NP 572075.1]; ORF 41: [GenBank: NP 572094.1]; ORF 29b: [GenBank: NP 572081.1]. **VZV ORFs**: ORF25: VOCs ID 59436; ORF65: 59475; ORF39: 59450; ORF60: 59470; ORF42: 59453.

## Supplementary Material

Additional file 1 **Supplementary Text**. The Supplementary Text [Additional file 1] gives full detail of the graph alignment method. The text further describes the methods used for sequence comparison and for calculations of statistical significance of the presented results. Click here for file

Additional file 2 **Supplementary Animation**. The Supplementary Animation [Additional file 2] illustrates the network alignment algorithm and shows the intermediate steps between the Figure 2a and Figure 2b. See caption of the Figure 2a,b for the colour coding of the nodes and links. Click here for file
