# Supplementary material for: From protein interactions to functional annotation: graph alignment in Herpes
Source: BMC Syst Biol. 2008 Oct 28;2:90. doi: 10.1186/1752-0509-2-90 (PMC2607256; doi:10.1186/1752-0509-2-90)
Supplement: Additional file 1 — Supplementary Text. The Supplementary Text [Additional file 1] gives full detail of the graph alignment method. The text further describes the methods used for sequence comparison and for calculations of statistical significance of the presented results. [file 1752-0509-2-90-S1.pdf]

Supplementary Text

Michal Kolář(1,2), Michael Lässig(1,3), Johannes Berg(1,3)
(1)Institut für Theoretische Physik, Universität zu Köln
Zülpicher Straße 77, 50937 Köln, Germany
(2)Institute of Molecular Genetics,
Academy of Sciences of the Czech Republic
Vídeňská 1083, 14220 Praha, Czech Republic
(3)Kavli Institute for Theoretical Physics,
University of California, Santa Barbara
CA 93106-4030 Santa Barbara, USA

July 22, 2008

Contents

1 Networks comparison **2**
1.1 Score parameters . . . 2
1.2 Consensus and pruned alignment . . . 3
1.3 Estimate of the p-value of the network alignment . . . 3

2 Sequences comparison **4**
2.1 Sequence score . . . 4

3 Graph alignment of VZV and KSHV **9**
3.1 Conservation of the network–aligned ORFs pairs at the sequence level . . . 9
3.2 Conserved links are more likely to be conserved across several herpesviral taxa . . . 11
3.3 Conserved links typically connect alike ORFs . . . 13

1 Networks comparison

The protein interaction data comes from the work of the group of Peter Uetz, [1], and is publicly available as the supplement of the cited article. The data are represented by a network (a graph) in which each node represents an open reading frame (ORF) of a species, and links denote experimentally observed protein interactions. Any network A is described by its adjacency matrix, which is a square matrix with terms a_{ij} equal 1 if there is an interaction between proteins i and j in the respective interaction network and zero otherwise. Graph alignment π is a mapping of nodes of a network A to nodes of a network B.

The graph alignment of the two networks has been performed as described in [2, 3]. The parameters were chosen in such a way that the algorithm remained in the high fidelity region. Scoring parameters are inferred from the actual data within the Bayesian approach described in [2]. The link and node contributions to the total score $S = S_L + S_N$ read:

$$\begin{aligned} S_L(\pi) &= \sum_{(ij)\in A^\pi} s_l(a_{ij}, b_{\pi(i)\pi(j)}) + \sum_{i\in A^\pi} s_s(a_{ii}, b_{\pi(i)\pi(i)}) \ , \\ S_N(s_1, s_2, \pi) &= \sum_{i\in A^\pi} \left[s_1(\theta_{i\pi(i)}) + \sum_{j\in B\setminus\pi(i)} w^\pi_{ij} s_2(\theta_{ij}) + \sum_{j\in A\setminus i} w^\pi_{j\pi(i)} s_2(\theta_{j\pi(i)}) \right] . \end{aligned} \quad (1)$$

Here we denote by A^π and B^π the subnetworks of the protein interaction networks A and B that are aligned by the network alignment π, $A \setminus i$ the set of all nodes in A but i. The sequence alignment score θ is defined in the Section 2. We further define the factor w^π_{ij} which prevents overcounting of score contributions. Its value is 1, when only one of i and j is aligned, and 0.5 when both nodes are aligned to different partners.

1.1 Score parameters

For the evaluation of the scoring parameters we accumulate the results of several runs of the alignment algorithm. We count the number of times m_{ij} a pair of nodes $i \in A$ and $j \in B$ were aligned in M runs of the algorithm, and set the corresponding density matrix term $\rho_{ij} = m_{ij}/M$. We can rewrite the definition in terms of the resulting alignments π^α,

$$\rho_{ij} = \frac{1}{M} \sum_{\alpha=1}^{M} \delta(\pi^\alpha(i), j) \ . \quad (2)$$

By $\pi^\alpha(i)$ we denote the alignment partner of the node $i \in A$ in the graph B in the run α. The term ρ_{ij} of the density matrix then approximates the probability of finding the pair (ij) in the final alignment.

For the evaluation of the link score matrices we count frequencies of matched/mismatched links in the alignment. That is, for each pair $(i, i') \in A$ and the alignment partners $j = \pi(i)$ and $j' = \pi(i')$ the terms $a_{ii'}$ and $b_{jj'}$ of the entries of the adjacency matrices are compared and the frequency table is accordingly updated. We calculate the frequency tables both for the links and the self-links:

$$\begin{aligned} q_l(a, b) &= \frac{1}{N_l} \sum_{(i,j)\in A} \sum_{(k,l)\in B} \rho_{ik}\rho_{jl}\delta(a_{ij}, a)\delta(b_{kl}, b), \\ q_s(a, b) &= \frac{1}{N_s} \sum_{i\in A} \sum_{k\in B} \rho_{ik}\delta(a_{ii}, a)\delta(b_{kk}, b) \ , \end{aligned} \quad (3)$$

where N_l and N_s are the normalisation constants of the two distributions and $a, b \in \{0, 1\}$.

If the two networks evolved independently, as it is assumed in the null model, we can marginalise the frequency tables and find the probabilities of having a link between two nodes in the graph A or B, $p^A_l(a) = \sum_{b=0}^{1} q_l(a, b)$, and $p^B_l(b) = \sum_{a=0}^{1} q_l(a, b)$. By the marginalisation of the self link distribution, we obtain p^A_s and p^B_s. Finally, we obtain the score parameters s_l and s_s by comparing the null and evolutionary model,

$$s_r(a, b) = \ln \frac{q_r(a, b)}{p^A_r(a) p^B_r(b)}, \quad r \in \{l, s\}. \quad (4)$$

Similarly, the node score parameters are inferred from the sequence similarities θ_{ij} and the current alignment. Three situations may occur for a pair of ORFs $i \in A$ and $j \in B$. Either the two ORFs are aligned in π according to their sequence homology, or their alignment contrasts the sequence homology (*i.e.*, sequence similar ORFs are not aligned to their homologs but to some other partners), or they are not aligned at all. These three disjoint sets of pairs of ORFs define three ensembles for which we evaluate frequencies of the sequence similarity θ; $d_1(\theta)$ for the aligned pairs, $d_2(\theta)$ for 'misaligned' pairs, and $d_0(\theta)$ for the pairs of nodes that are not aligned. We take the score θ as defined in the Section 2 as the sequence similarity measure. The three distributions of θ are

$$d_1(\theta) = \frac{1}{N_1}\sum_{i\in A}\sum_{j\in B}\rho_{ij}\theta_{ij}\delta(\theta-\theta_{ij}), \quad (5)$$

$$d_2(\theta) = \frac{1}{N_2}\sum_{i\in A}\sum_{j\in B}(1-\rho_{ij})\left[1-\prod_{k\in A,k\neq i}(1-\rho_{kj})\prod_{l\in B,l\neq j}(1-\rho_{il})\right]\theta_{ij}\delta(\theta-\theta_{ij}), \quad (6)$$

$$d_0(\theta) = \frac{1}{N_0}\sum_{i\in A}\sum_{j\in B}(1-\rho_{ij})\prod_{k\in A,k\neq i}(1-\rho_{kj})\prod_{l\in B,l\neq j}(1-\rho_{il})\theta_{ij}\delta(\theta-\theta_{ij}) \ , \quad (7)$$

where N_0, N_1, and N_2 are normalisation constants. In general, we expect $d_1(\theta)$ to be an increasing function of θ, reflecting the fact that the aligned ORFs should have similar functions. Indeed, many sequence–homologous pairs belong to this set. The distribution $d_2(\theta)$ is, on the other hand, expected to be a decreasing function of θ, similarly to $d_0(\theta)$.

The distribution $d_0(\theta)$ of similarities of unaligned ORFs may be considered as the background distribution of θ, and is taken as the distribution in the null model. The node scores s_1 and s_2 read

$$s_r(\theta) = \ln\frac{d_r(\theta)}{d_0(\theta)}, \quad r \in \{1,2\}. \quad (8)$$

1.2 Consensus and pruned alignment

From the ρ matrix we extract the consensus alignment as the alignment of ORFs that have the corresponding ρ-matrix term larger than 0.5. The consensus alignment is then pruned in order to remove marginally aligned pairs. These we define as the pairs that have a negative sequence score and at the same time less than two matching interactions. This pruning removes spuriously aligned pairs with both low sequence similarity and low topological match.

1.3 Estimate of the p-value of the network alignment

To calculate the p-value of aligning two nodes $i \in A$ and $j \in B$, we remove the pair (ij) from the alignment and find the probability of placing in the vacancy a pair of nodes with a topological match as good or better than the match of the pair (ij). These two nodes are chosen from two Erdős–Rényi networks with sizes and mean connectivities identical to those of the KSHV and VZV networks (the null model of independently evolved networks).

A pair of nodes has the same or better topological match whenever it has the same or a larger number of matching links to other aligned pairs or it has a smaller number of mismatching links. For the pair (ij) with r matching links in the alignment graph (Figure 2a in the main text) the p-value is defined as the probability of finding a nodes pair with r or more matching links and at most $n_A - r$ ($n_B - r$ respectively) mismatching links, where n_A (n_B) is the total number of links adjacent to i in A^π (j in B^π).

This probability is easily evaluated for uncorrelated networks using the multinomial distribution. For $p_A \ll 1$ and $p_B \ll 1$ it reads

$$\begin{aligned} p(r,n_A,n_B,N^\pi) = & \quad (9)\\ & (N_A - N^\pi + 1)(N_B - N^\pi + 1) \\ & \times \sum_{m_A=r}^{n_A}\sum_{m_B=r}^{n_B}\sum_{s=r}^{s=\min\{m_A,m_B\}}\binom{N^\pi - 1}{s, m_A - s, m_B - s, N^\pi - 1 - m_A - m_B + s} \\ & \times [p_A p_B]^s [p_A(1-p_B)]^{m_A - s}[(1-p_A)p_B]^{m_B - s}[(1-p_A)(1-p_B)]^{N^\pi - 1 - m_A - m_B + s} \ , \end{aligned}$$

where N^π is the size of the aligned subnetworks ($N^\pi = 26$), and p_A and p_B are the link probabilities in the two Erdős–Rényi graphs which we estimate from the complete KSHV and VZV networks respectively, giving $p_A = 0.0330$ and $p_B = 0.0561$. The individual terms of equation (9) can be understood intuitively: first we choose a node in the network $A \setminus A^\pi \cup i$ (one node out of $N_A - N^\pi + 1$), and a partner node from the network $B \setminus B^\pi \cup j$. Next we choose from the $N^\pi - 1$ remaining nodes in the alignment network s nodes that are connected by matching links with the probability $p_A p_B$, $m_A - s$ ($m_B - s$ respectively) nodes that are connected by links only in the KSHV (VZV) subnetwork with appropriate probability, and the remaining nodes that are not linked to the pair (ij) in either subnetwork. Finally, we sum over all possible choices of the nodes (the multinomial coefficient) and over all options that are equally good or better than the actual alignment of (ij). The contribution from the self–links (which are typically mismatching) is close but smaller than 1 and is neglected here. The result is then an upper bound of the p-value. The estimated p-values for the pairs of ORFs discussed in the main text are listed in the Table 4.

Similarly, we estimate the p-value of finding in the alignment networks a clique with M_C pairs, out of which M_O pairs are sequence related, and which are connected by matching links only. We calculate this p-value as the probability of finding such a clique and of finding among the links adjacent to the vertices of the clique the same number or more matching links and the same number or less of links that are present in one protein interaction network only. Denoting the pairs of the clique $(i^a j^a)$, where $a \in \{1, 2, \ldots, M_C - M_O\}$, the numbers of the matching links r^a, and the total number of links adjacent to i^a (j^a) in KSHV (VZV) as n_A^a (n_B^a), this p-value is

$$
\begin{aligned}
& p(M_C, M_O, \{r^a\}, \{n_A^a\}, \{n_B^a\}, N^\pi) = \qquad (10) \\
& \quad \binom{N_A - N^\pi + M_C - M_O}{M_C - M_O} \binom{N_B - N^\pi + M_C - M_O}{M_C - M_O} (M_C - M_O)! (p_A p_B)^{\binom{M_C}{2}} \\
& \quad \times \prod_{a=1}^{M_C - M_O} \sum_{m_A = r^a}^{n_A^a} \sum_{m_B = r^a}^{n_B^a} \sum_{s = r^a}^{s = \min\{m_A, m_B\}} \binom{N^\pi - M_C}{s, m_A - s, m_B - s, N^\pi - M_C - m_A - m_B + s} \\
& \quad \times [p_A p_B]^s [p_A(1 - p_B)]^{m_A - s} [(1 - p_A) p_B]^{m_B - s} [(1 - p_A)(1 - p_B)]^{N^\pi - M_C - m_A - m_B + s} .
\end{aligned}
$$

The contribution of self links is again omitted and giving upper bound to the p-value. The formula (10) reduces to (9) in the case of an isolated node (1-clique) in which case $M_C = 1, M_O = 0$.

The p-value for the clique formed by the pairs 67.5/25, 28/65, 29*b*/42, 23/39 given by (10) is 5×10^{-11}. The p-values of finding such a clique in the protein interaction networks of the two species can be estimated similarly and they are 2×10^{-3} in KSHV and 4×10^{-2} in VZV. The difference between the p-value inferred from the aligned networks and the p-values estimated from the single–species networks indicates the significance of the evolutionary conservation of the clique.

2 Sequences comparison

The sequences of the two herpesviruses (KSHV strain BC-1 and VZV Oka-parental) have been downloaded from the Viral Orthologous Clusters (VOCs) database [4]. Further ORFs (transcript variants) have been obtained from the NCBI database [5] or the VIDA virus database [6] or have been provided by Peter Uetz [1].

To assess mutual sequence similarity of the ORFs in the two viral species we generate sequence alignments of each KSHV ORF with each VZV ORF. Since the open reading frames are short and the level of sequence similarity is low, care has to be taken in obtaining the optimal alignment, as detailed below.

To account for the uneven level of sequence conservation across the genome, we optimise the scoring parameters of the Needleman–Wunsch algorithm individually for each pair of ORFs [7]. We optimise the following parameters: the gap–opening penalty, the gap–extension penalty and the evolutionary distance encoded by the BLOSUM matrices, [8]. The code for the sequence alignment, termed *sequenceAlign* is available upon request.

2.1 Sequence score

We define a standard log-likelihood score of an alignment of two sequences by comparing a model based on evolutionary relation of the two sequences with a random model. The random model of independently evolved sequences depends only on the frequencies of amino-acids occurring in natural

peptides. If we denote these frequencies by $p(a)$, where a stands for an amino-acid residue and has 20 possible values, we may write the probability of generating randomly a sequence $\mathbf{a}$ of length L with a composition $\{a_i\}$ as

$$P(\mathbf{a}) = \prod_{i=1}^{L} p(a_i) \ . \tag{11}$$

The probability of generating sequences $\mathbf{a}$ and $\mathbf{b}$ under this uncorrelated model reads

$$P'(\lambda, \mathbf{a}, \mathbf{b}) = P(\mathbf{a})P(\mathbf{b}) = \prod_{j=1}^{L} p(a_j)p(b_j) \ . \tag{12}$$

For evolutionary related sequences, we expect a higher probability observing two equal or similar residues, which is expressed by the log-likelihood score matrices σ. Hence

$$Q'(\lambda, \mathbf{a}, \mathbf{b}) = \frac{1}{Z'(\mathbf{a}, \mathbf{b})} \prod_{j=1}^{L} p(a_j)p(b_j)e^{\sigma(a_j, b_j)} \ , \tag{13}$$

where $Z'(\mathbf{a}, \mathbf{b})$ is a normalisation constant

$$Z'(\mathbf{a}, \mathbf{b}) = \sum_{\lambda} \prod_{j=1}^{L} p(a_j)p(b_j)e^{\sigma(a_j, b_j)} \ . \tag{14}$$

The construction of the scoring matrices of the BLOSUM series ascertains that the normalisation constant Z' equals 1 for sequences with the residue frequencies $p(a)$ close to those inferred from current databases. This condition is also typically satisfied for all proteins with 100 and more residues. The log-likelihood score of an alignment (without gaps) is then expressed as

$$\begin{aligned} \theta'(\lambda, \mathbf{a}, \mathbf{b}) &= \ln \frac{Q'(\lambda, \mathbf{a}, \mathbf{b})}{P'(\lambda, \mathbf{a}, \mathbf{b})} \\ &= \sum_{j=1}^{L} \sigma(a_j, b_j) - \ln Z'(\mathbf{a}, \mathbf{b}) \ . \end{aligned} \tag{15}$$

With the proper normalisation of Q' by Z', the score θ' is larger than zero whenever the two sequences $\mathbf{a}$ and $\mathbf{b}$ are more likely to evolve under the evolutionary model underlying the scoring matrices in use.

To allow gaps in the global alignment we add two more parameters to the model, the gap–opening penalty $\ln \mu$ and the gap–extension penalty $\ln \nu$ (affine gaps). The score splits into two parts: the substitutions score and the gap score:

$$\begin{aligned} \theta(\lambda, \mathbf{a}, \mathbf{b}, \mu, \nu, \sigma) &= \ln \frac{Q(\lambda, \mathbf{a}, \mathbf{b})}{P(\lambda, \mathbf{a}, \mathbf{b})} \\ &= \sum_{\text{aligned r.}\, j} \sigma(a_j, b_j) + \sum_{\text{gaps}\, j} [\ln \mu + (l_j - 1) \ln \nu] - \ln Z^L \ . \end{aligned} \tag{16}$$

Here we first sum all contributions from residue substitutions and then we sum all the gap costs. The affine gap costs increase linearly with the gap length l_j.

Z^L is the normalisation constant of the probabilities Q and it depends on the length of the alignment L, the two sequences, the scoring matrix in use, and the gap score parameters. Since the BLOSUM score matrices are properly normalised by construction, $Z' = 1$, or

$$\sum_{a,b} p(a)p(b)e^{\sigma(a,b)} = 1 \ , \tag{17}$$

the only contribution to Z^L comes from the gaps. To calculate this contribution, we will consider the following Markov chain.

We start with the two sequences completely unaligned and we choose one option of: either *(i)* we align the two initial residues of the considered sequences (a substitution), or *(ii)* we align the initial residue of the second sequence with a gap, that is, we create a gap on the first sequence (a

deletion), or *(iii)* we create a gap on the other sequence (an insertion). In this way the alignment is started and we extend it by one of the following steps: either *(i)* we align the residues that follow in the two sequences (a substitution), or *(ii)* we create the gap on the first sequence (a deletion), or *(iii)* we create a gap on the other sequence (an insertion). We repeat the steps *(i–iii)* until the last residue is aligned to a residue or to a gap. The length of the alignment L is the number of the steps in the Markov chain. For this Markov chain we can calculate the normalisation constant Z^L by a simple transfer matrix method. At each step l there are three possibilities of the end state of the alignment: either the last step was a substitution, or a deletion or an insertion. Hence, we split Z^l in three parts $Z^l = Z^l_s + Z^l_d + Z^l_i$ that correspond to the respective end-states. We may express the vector $Z^{l+1} = (Z^{l+1}_s, Z^{l+1}_d, Z^{l+1}_i)$ at step $l+1$ of the Markov chain as a function of the vector Z^l at the step l:

$$Z^{l+1} = TZ^l, \tag{18}$$

where the transfer matrix T reads

$$T = \begin{pmatrix} 1 & 1 & 1 \\ \mu & \nu & 0 \\ \mu & 0 & \nu \end{pmatrix}. \tag{19}$$

At the beginning of the alignment process we may start with a substitution, a deletion or an insertion and hence $Z^0 = (1,1,1)$. The normalisation constant for an alignment of length L can be readily calculated by applying the transfer matrix L-times on the initial vector, $Z^L = T^L Z^0$. For long alignments the dominant contribution comes from the largest eigenvalue of the transfer matrix and it reads

$$Z^L = \frac{(2\mu - \nu + 3\alpha)}{\sqrt{(\nu-1)^2 + 8\mu}} \alpha^L \ . \tag{20}$$

Since the logarithm of the normalisation constant $\ln Z^L = C(\mu,\nu) + L \ln \alpha$ is extensive in the length L and since L is the sum of numbers of substitutions, deletions and insertions, the normalisation can be implemented as a shift of scores:

$$\begin{aligned} \theta(\lambda, \mathbf{a}, \mathbf{b}, \mu, \nu, \sigma) &= \sum_{\text{aligned r.}} (\sigma(a_j, b_j) - \ln \alpha) \\ &+ \sum_{\text{gaps}} [\ln \mu - \ln \alpha + (l_j - 1)(\ln \nu - \ln \alpha)] - C(\mu, \nu). \end{aligned} \tag{21}$$

The score defined by the last formula is properly normalised for any choice of scoring parameters μ, ν and σ, whenever the substitution scoring matrix is normalised according to (17). The normalisation is done against all alignments of length L, what is an approximation of the exact normalisation evaluated by Yu and Hwa, [9], who considered all possible alignments of the two sequences. However, this approximation allows to evaluate the normalisation constant explicitly (instead of the iterative formulae of [9]) and is at the same time a very good estimate for sufficiently large negative gap penalties. The normalisation allows us to search for the optimal parameters for an alignment of any two sequences $\mathbf{a}$ by maximising the score $\theta(\lambda, \mathbf{a}, \mathbf{b}, \mu, \nu, \sigma)$ over its arguments: the alignment λ and the parameters μ, ν, σ. This maximisation is performed iteratively by the code *sequenceAlign*.

The final score is computed by subtracting the contribution of leading and trailing gaps. All alignments which are either too short (6 residues and less) or contain too many gaps (a gap opening every 6th residue on average), are disregarded as insignificant. The final score is used as the measure of the sequence similarity θ which is used, in completion to interaction data, in the network alignment. For the remaining alignments we compute also the percent identity defined as the number of identities in the alignment divided by the total number of substitutions in the alignment. Knowing the optimal alignment and its score for all pairs of nucleotide sequences, we search for the reciprocally best matching ORFs in the two species, considered bona-fide *sequence homologs*.

The number of sequence homologs in the KSHV/VZV genome is 34, that is approximately 40% of the ORFs of each species. The list of the sequence homologs and parameters of their alignments are given in the Table 1, together with the scores calculated using *clustalW* (version 1.81, default parameters [10]). For the four ORFs pairs discussed in the Results section of the main text we have estimated also p-values of the *clustalW* alignment and we present the data in the Table 3. The *sequenceAlign* scores are directly comparable with the *clustalW* scores and the obtained alignments differ only marginally, see Figure 1 for an example.

a) *sequenceAlign*

```
Query=  KSHV-BC1-ORF67.5            Length= 80
Sbjct=  VZV-Oka_p-ORF25             Length= 156
Score=  0.2433
logMu  -6.798, logNu 0.006, logAlpha 0.049,
Matrix: blosum50
%
%Q:                EYAS-----------------------------------------------------
%S:                YESENASEHHPELEDVFSENTGDSNPSMGSSDSTRSISGMRARDLITDTDVNLLNIDALE
%                    +

Q:                ----------------DQLLPRDMQILFPTIYCRLNAINYCQYLKTFLVQR--------A
S:                SKYFPADSTFTLSVWFENLIPPEIEAILPTTDAQLNYISFTSRLASVLKHKESNDSEKSA
                                  ++L+P +++ ++PT   +LN I++ + L + L ++        A

Q:                QPAACDHTLVLESKVDTVRQVLRKIVSTDAVFSEA
S:                YVVPCEHSASVTRRRERFAGVMAKFLDLHEILKDA
                      C+H+  +  + +    V+ K +    ++ +A
```

b) *clustalW*

```
Sequence 1: KSHV-BC1-ORF67.5               80 aa
Sequence 2: VZV-Oka_p-ORF25                156 aa
Alignment Score 57
CLUSTAL W (1.81) multiple sequence alignment

KSHV-BC1-ORF67.5  --------MEYAS----DQLLPRDMQILFPTIYCRLNAINYCQYLKTFLVQRAQP-----
VZV-Oka_p-ORF25   ESKYFPADSTFTLSVWFENLIPPEIEAILPTTDAQLNYISFTSRLASVLKHKESNDSEKS
                            ++     ++L+P +++ ++PT  ++LN I++ + L ++L ++ +

KSHV-BC1-ORF67.5  ---AACDHTLVLESKVDTVRQVLRKIVSTDAVFSEARARP
VZV-Oka_p-ORF25   AYVVPCEHSASVTRRRERFAGVMAKFLDLHEILKDA----
                     ++C+H+  +  + + +  V+ K+++ + ++++A
```

Figure 1: **Comparison of performance of *sequenceAlign* and *clustalW*** Sequence alignment of distantly related ORFs KSHV 67.5 and VZV ORF 25. Both algorithms find alignments with 20% (18%) identity over approximately 80 aa. The score of the *sequenceAlign* alignment is 0.2, meaning that the random model is almost as likely as the model of evolutionary related sequences. This is also shown by the very high p-value of the *clustalW* alignment, $p = 0.44$.

KSHV ORF	VZV ORF	seq. orth.	*sequenceAlign* identity (%)	score	*clustalW* identity (%)	score
9	28	*	43.3	467	39.8	2155
70	13	*	63.7	369	61.8	1247
44	55	*	36.5	293	34.1	1431
25	40	*	29.9	281	27.0	1628
61	19	*	32.6	174	31.2	1035
60	18	*	37.4	162	37.6	676
29b	42	*	38.4	148	37.8	680
8	31	*	24.8	145	24.3	924
29b	45		41.2	123	35.3	643
46	59	*	39.9	113	43.4	560
43	54	*	24.5	89	24.5	668
6	29	*	20.1	81	20.6	790
56	6	*	30.2	51	23.3	726
7	30	*	23.6	48	22.4	548
68	26	*	20.0	34	20.7	370
29a	45		28.5	34	25.1	305
39	50	*	18.9	32	18.6	280
37	48	*	23.5	32	19.8	291
20	35	*	34.2	22	23.0	154
17	33	*	28.1	22	21.9	311
19	34	*	19.8	10	18.1	304
53	9a	*	23.8	8	28.6	76
26	41	*	14.5	4	20.3	169
67.5	25	*	20.0	0	18.4	57
28	65	*	9.9	0	10.8	-31
53	8.5		20.8	-1	26.4	57
K6	1	*	10.6	-1	11.6	-26
69	27	*	21.7	-1	16.4	126
28	8.5		20.0	-1	14.7	-41
67	24	*	14.0	-3	14.3	2
30	8.5		16.2	-4	20.8	6
72	7	*	10.2	-7	13.4	-42
52	1		13.1	-8	18.5	7
65	0	*	20.6	-9	18.2	-19
38	49	*	21.7	-9	18.0	11
72	35		6.6	-9	13.3	-38
28	1		11.9	-10	14.7	-29
53	0		13.9	-11	17.3	31
52	46		17.7	-11	19.9	27
K6	S/L		11.7	-11	20.0	5
30	9a		14.5	-11	19.5	20
53	65		6.9	-11	15.7	-17
67.5	49		7.6	-12	11.7	-31
K5	58	*	11.4	-12	12.7	-48
67.5	9a		15.4	-12	15.6	-19
38	7		11.7	-12	29.5	16
K15	65		8.1	-12	10.1	-42
16	69	*	11.5	-12	10.9	-42
16	64	*	11.5	-12	10.9	-42
67.5	7		13.9	-13	25.0	6
K8	23	*	10.7	-13	13.9	-18
K4	S/L		12.9	-13	22.3	18
K4	9a		4.7	-13	19.2	1
53	1		4.7	-14	14.2	-28
30	57		10.0	-14	17.4	-23
74	36	*	10.0	-14	12.9	-30
55	58		9.1	-14	9.6	-93

Table 1: **The detected sequence homologs:** We list all the pairs of putative sequence homologs detected by *sequenceAlign* together with the score and the percent identity returned by the code. The score and the percent identity obtained with *clustalW* (version 1.81, standard parameters values) are also listed. The pairs that are considered putatively sequence homologous are marked by asterisk.

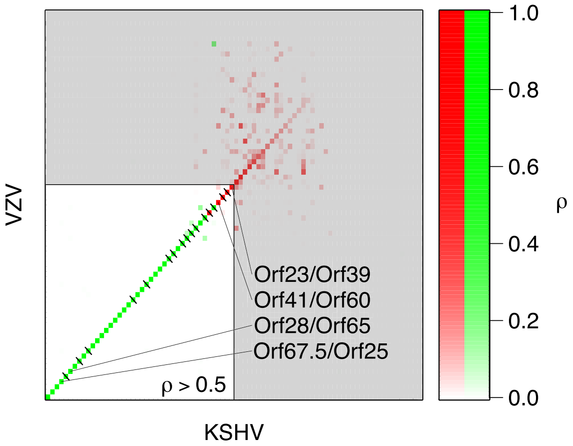

Figure 2: **The probing of the 'twilight zone' of low and no sequence similarity by the alignment:** The noise parameter of the algorithm is set just before the onset of the low-fidelity regime ($T = 5$). The alignment is represented by the matrix ρ, with $\rho(i, j)$ indicating the relative frequency with which a node i is aligned with j over many alignment runs. Entries of ρ coloured green correspond to node pairs with mutual sequence similarity, those coloured red have no sequence similar partner and are thus aligned on the basis of link similarity alone. The conservative consensus alignment with $\rho > 0.5$, and aligned node pairs with less than two matching links discounted (crossed-out points), is at the bottom left. At lower values of ρ spurious alignments occur (top right). The marked cases yield functional predictions discussed in the Main text.

3 Graph alignment of VZV and KSHV

The optimal temperature for the algorithm run has been estimated from the comparison with randomly generated data, see [3] for details. In this way, we maximise the number of aligned pairs while aiming to keep the estimated number of wrongly aligned pairs negligible. The alignment contains 26 node pairs out 84 of KSHV and 76 of VZV (approximately 33%).

The list of pairs of ORFs that are present in the resulting alignment is shown in the Table 2 together with local scores for the pairs. The local scores give the contributions of the pair to the total node and link scores of the alignment. We further represent the alignment in the Figure 2 by the ρ matrix as defined in Section 1.1.

Comparison of the sequences of the pairs of ORFs which are discussed in the Results section of the main text are summarised in the Table 3. The comparison of the interaction patterns of these pairs is summarised in the Table 4.

Together with other characteristics of the aligned ORFs (the sequence length and the position in the genome described in the main text), we compared also the GC content of the aligned pairs. The plot in the Figure 3 shows that there is no correlation of this sequence characteristic. The fact that also very closely related herpesviral ORFs may have very different GC contents has been observed already by Vlček *et al.* in [11].

3.1 Conservation of the network–aligned ORFs pairs at the sequence level

The pairwise sequence comparison described in Section 2 have not yielded a significant sequence similarity for 2 node pairs aligned solely due to their interaction similarity. To further test the possibility of detection of sequence homology we have searched for multiple sequence alignments of the protein families to which these ORFs belong. We have extracted the respective families from the VOCs database [4], and compared them using *DIALIGN* [12], *Parallel PRRN* [13], *MUSCLE* [14], *T-COFFEE* [15], *PSALIGN* [16], *SAM-T99* [17], and *MSA* [18].

For each pair KSHV 67.5/VZV 25, 28/65, 23/39, 41/60 we have selected from the VOCs database a representative subset of the herpesviral proteins in the same family (at least ten or all proteins) and compared these families using the multiple alignment searching tools. While for the pair 67.5/25 we have found very weak alignment[1] of the corresponding families, for the other three pairs we

[1]T-Coffee alignment has two stretches of more than 20 aa with $CORE > 3$ (T-Coffee 5.05 EMBL-EBI, default configuration).

KSHV ORF	VZV ORF	node score	link score
28	65	3.50	6.30
29b	42	4.30	6.14
67.5	25	4.20	4.57
23	39	-0.49	4.47
41	60	-0.49	4.39
61	19	5.41	2.00
60	18	5.41	1.67
9	28	5.41	0.91
6	29	5.41	0.35
25	40	5.41	0.35
37	48	5.41	0.35
20	35	4.66	0.35
29a	45	4.30	0.35
43	54	5.41	0.35
70	13	5.41	0.35
8	31	5.41	0.35
7	30	5.41	0.14
44	55	5.41	0.14
19	34	5.41	0.06
56	6	5.41	0.01
53	9a	2.49	-0.08
17	33	5.41	-0.16
39	50	5.41	-0.29
26	41	5.21	-0.29
46	59	5.41	-0.29
68	26	5.41	-0.76

Table 2: **The list of ORFs in the optimal alignment.** The aligned node pairs are ordered according to the value of the link score.

KSHV ORF	VZV ORF	seq. orth.	***sequenceAlign***			***clustalW***			
			identity (%)	length	score	identity (%)	length	score	p-value
67.5	25	*	20.0	80	0	18.4	76	57	0.44
28	65	*	10.8	102	0	10.8	102	-31	0.66
23	39		—	—	—	17.5	240	41	0.43
41	60		—	—	—	11.9	160	-42	0.94

Table 3: **The sequence similarity of the pairs that are discussed in the Result Section of the main text.** Results of *sequenceAlign* and *clustalW* are shown. The p-values for the *clustalW* results are calculated from the ensemble of randomised sequences.

KSHV ORF	VZV ORF	links in the alignment		shared links	link score	p-value
		KSHV	VZV			
67.5	25	5	12	4	4.57	4×10^{-3}
28	65	4	5	4	6.30	1×10^{-3}
23	39	4	4	3	4.47	2×10^{-2}
41	60	3	6	3	4.39	2×10^{-2}

Table 4: **Topological similarity of the pairs aligned because of conservation of protein interaction network topology.** The numbers of common links and other links in the aligned subnetwork of the KSHV and VZV network are listed, together with the resulting link score. The p-values are given by equation (9).

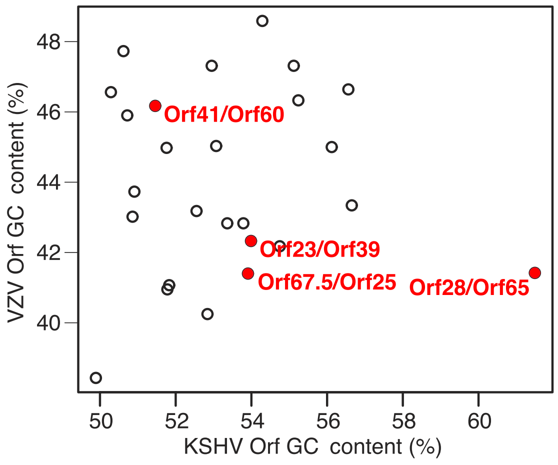

Figure 3: **GC content analysis does not show any correlation.** The correlation of GC content has decayed during the independent evolution of the two viruses.

have detected no sequence similarity. This observation further shows the extend of the evolutionary divergence for the pairs of ORFs.

Returning back to the pairwise alignment we have generated the dot plots for the pairs listed in the Table 1. Here we observe a very clear pattern: while for the ORFs pairs with a high similarity the dot plots are dominated by a single diagonal, with increasing divergence this diagonal disappears among short diagonal lines that correspond to random alignments, see Figure 4. The network–aligned pairs show the dot–plot pattern of an intermediate quality.

3.2 Conserved links are more likely to be conserved across several herpesviral taxa

Conservation of an interacting pair of proteins (an interolog) between KSHV and VZV raises the question if an interolog is present also in other species of herpesviruses. For this purpose, we have used data on protein interaction networks of HSV (HHV-1, α-herpesviridae), mCMV (murine cytomegalovirus, closely related to human cytomegalovirus, β-herpesviridae) and EBV (Epstein–Barr virus, γ-herpesviridae) kindly provided by Peter Uetz laboratory (data not published), and the protein interaction data of EBV published by Calderwood *et al.*, [19]. All the data have been obtained by yeast–two–hybrid assays and are, hence, comparable to the protein interaction networks of KSHV and VZV.

In the analysis, we concentrate on the VZV protein interaction network, since the statistics for the KSHV network turns out to be insignificant due to a small number of interologs in the dataset. To answer the question, we compare the likeliness of being conserved for the links of VZV that are inside the alignment and the links that are not in the alignment π. We classify the link to be outside of the alignment if both adjacent nodes are not aligned by π. For such links we do not expect to find an interolog in other herpesviral species while we expect to find interologs in the other herpesviral species for the links within the alignment. To measure the likeliness of a link to have an interologs, we first define a weighted network M that represents the three interaction networks of HSV, mCMV, and EBV and their relation to the network of VZV. The network M is constructed in the following way: taking all pairs of nodes (i, j) of the VZV network V, we search for interologs in EBV, HSV, and mCMV protein interaction networks. A pair of interacting proteins is considered interologous when both partners have BLAST (default parameters) expectation value smaller than 0.05 when searched against complete genome of the other species. If such an interolog exists in a single species, we connect the nodes i and j with a link m_{ij} with weight 1/3. If the interolog exists in exactly two species, we put a link with weight 2/3. If the interolog exists in all three networks, the link assumes weight 1. And finally, if no interolog exist, we put $m_{ij} = 0$—the link does not exist in M. Thus each weight m_{ij} in M provides information on whether there is an interolog of (i, j) in EBV, HSV, or mCMV, and also how many such interologs exists.

To evaluate the likeliness of an interaction to be conserved we define the frequency tables q^{π} and q^{out}. The frequency table q^{π} is defined as a sum over all node pairs within the aligned subnetwork

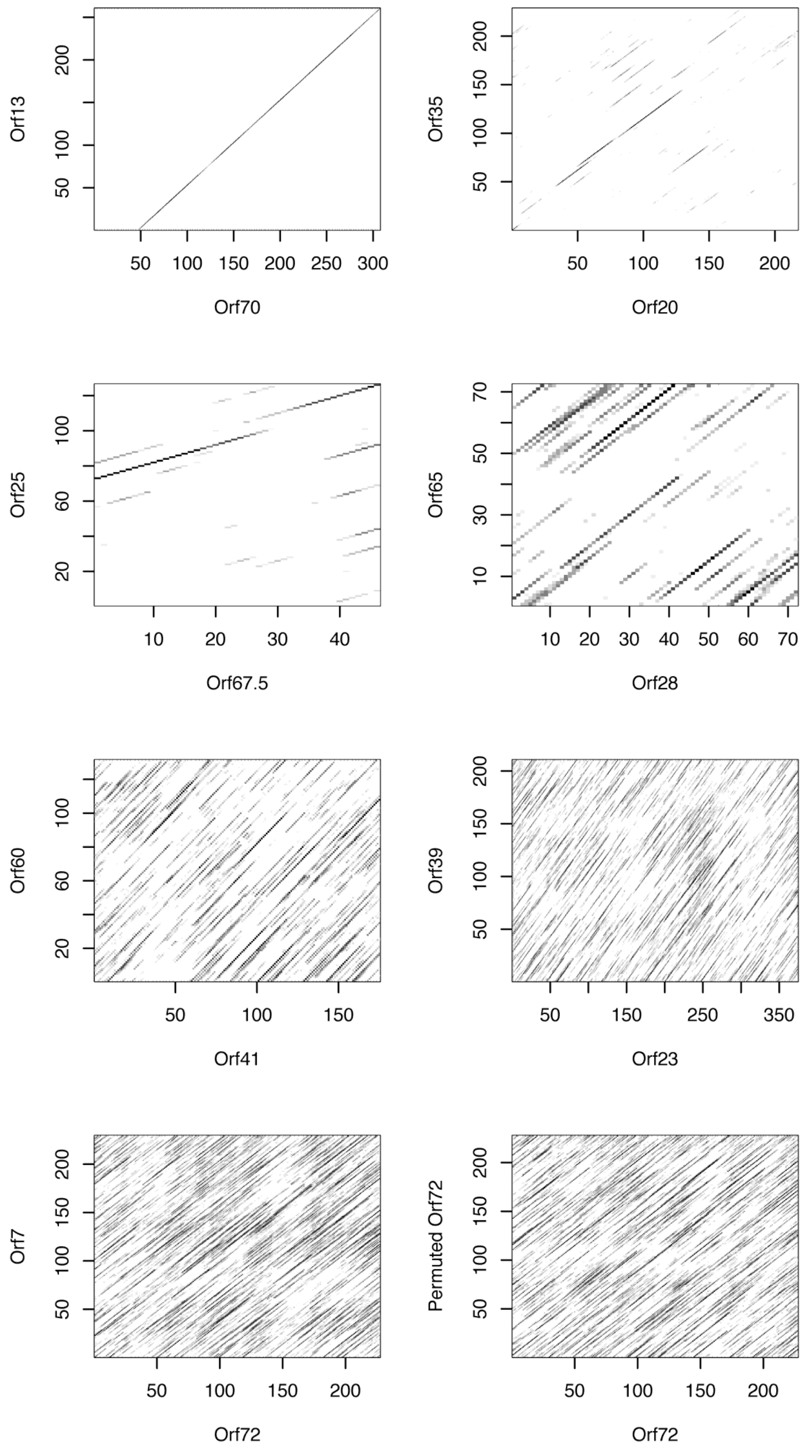

Figure 4: **Network alignment allows detection of homologs with poor sequence similarity**. With decreasing level of sequence conservation the dominant diagonal in the dot plot disappears among traces of random alignments. From top–left to bottom–right: ORFs KSHV 70/VZV 13, an almost perfect match; ORFs 35/20, a typical match of sequence homologs, ORFs 67.5/25, the pair aligned due to sequence and network conservation; ORFs 28/65, 41/60 and 23/39, the pairs aligned dominantly or only because of interaction conservation; ORFs 72/7, spurious sequence homologs not aligned by the network alignment; ORF 72/permuted ORF 72, comparison with a random sequence. The sliding window of size 30 has been used for the generation of the dot plots.

of V,

$$q^{\pi}(g,h) = \frac{\sum_{(i,j)\in V^{\pi}} [\delta(v_{ij},g)\,\delta(m_{ij},h)\,m_{ij} + \delta(v_{ij},g)\,\delta(m_{ij},1-h)\,(1-m_{ij})]}{\sum_{(i,j)\in V^{\pi}} 1}, \tag{22}$$

while $q^{out}(i,j)$ is defined similarly as a sum over all node pairs outside of the alignment. The mutual informations I^{π} and I^{out} give the measure of likeliness of an interaction to have interologous interactions in the other herpesviral networks:

$$I^{\pi} = \sum_{(g,h)} q(g,h) \ln \frac{q(g,h)}{q^{V}(g)q^{M}(h)}. \tag{23}$$

The marginal q^V is calculated by marginalisation of $q(g,h)$ over h, and q^M by marginalisation over g. We repeat the same calculation for I^{out}.

We find in the aligned network of VZV 7 out of 35 links interologous to some other herpesviral species, while in the 'outside' network we find 5 out of 53 links interologous. These figures lead to mutual informations $I^{\pi} = 3\times10^{-3}$ and $I^{out} = 4\times10^{-4}$. The difference expressed by the odds ratio $L = I^{\pi}/I^{out}$ equals 6.6. To evaluate the significance of the result, we have generated 1000 random alignments of KSHV and VZV, with the same number of nodes as the actual subnetwork V^{π}, repeated the analysis and obtained the p-value 0.3. When, instead of the mutual informations, Pearson correlations C^{π} and C^{out} are taken as the measures of conservation, we get $C^{\pi} = 0.089$ and $C^{out} = 0.035$. Their ratio $L' = C^{\pi}/C^{out}$ equals 2.5.

The interactions among the aligned proteins of KSHV and VZV are thus more likely to have an interolog in other species of herpesviruses.

3.3 Conserved links typically connect alike ORFs

To examine the relationship between function of the proteins and the conservation of links among them, we analyse the likeliness of the conservation of the links among the proteins with similar functions and of the conservation of the links between the proteins with dissimilar functions.

First we test if the conserved links are more likely to connect alike proteins. To do so, we group the ORFs to two functional classes: the protein belongs either to the class of 'structure–related' proteins (classes: capsid/core protein, membrane/glycoprotein, virion protein, virion assembly) or to the class of 'information–processing' proteins (DNA replication, gene expression regulation, nucleotide repair/metabolism, host–virus interaction). We calculate the frequencies $p(f_i, f_j)$ of functional annotations f_i, f_j of adjacent ORFs i and j in the subgraph containing all sequence homologs (resp. all network–aligned ORFs),

$$p(g,h) = \frac{\sum_{links} \delta(f_i,g)\delta(f_j,h)}{\sum_{links} 1}. \tag{24}$$

We evaluate this sum separately for all the links in the subgraph (p_A), the conserved links only (p_M) and for the nonconserved (mismatching) links in the subgraph (p_{MM}).

Then we calculate the mutual information as the measure of the correlation of the functional annotation of the adjacent ORFs,

$$I = \sum_{g}\sum_{h} p(g,h) \ln \frac{p(g,h)}{p(g)p(h)}, \tag{25}$$

where $p(g)$ is the marginal $p(g) = \sum_h p(g,h)$. Keeping the subscripts we find: $I_A = 0.0014$ for the subgraph of sequence homologs (0.0092 for the alignment subgraph), $I_M = 0.0743$ (0.1178), and $I_{MM} = 1\times10^{-6}$ (0.0001). Clearly, the greatest mutual information on functional annotation of adjacent ORFs is among nodes connected by matching links (by a factor of 100 or more). These correlations, when expressed in terms of Pearson correlations C_A, C_M, and C_{MM} read $C_A = 0.054$, $C_M = 0.381$, and $C_{MM} = -0.002$. Clearly, the functions of ORFs connected by mismatched links are not correlated. We have evaluated also the frequency tables for the complete protein interaction networks, p_K and p_V. Not surprisingly, the mutual information is small for these graphs $I_K = 0.0030$, and $I_V = 0.0052$ (Pearson correlations $C_K = 0.077$ and $C_V = 0.103$).

To estimate the p-value of such a mutual information we have reshuffled the positions of the conserved links randomly and we have evaluated the mutual information I_M for such randomised graphs. The probability of finding equal or better mutual information I_M in an ensemble of 10^5

graphs generated in this way has been taken as the p-value. The estimates are 0.12 for the subgraph of sequence homologs and 0.05 for the alignment subgraph.

Secondly, we test if the links between similar proteins are more likely to be conserved. Taking the subgraph of the sequence homologs as the basis of our analysis, we create the matrix $n_F(a,b)$ defined in the following way: $n_F(0,0)$ is the number of pairs of the alike ORFs between which there is a link in neither species; $n_F(1,0)$ is the number of the pairs of the alike ORFs that are connected by a link in KSHV solely; $n_F(0,1)$ the same for VZV; and $n_F(1,1)$ is the number of conserved links between alike ORFs. We create the second matrix n_D defined similarly for the pairs of ORFs with unlike functional annotation.

Then we define the conservation ratio p_F as the ratio of the number of conserved links and the total number of links

$$c_F = n_F(1,1)/(n_F(0,1) + n_F(1,0) + n_F(1,1)). \tag{26}$$

In the same way we define c_D for the links between unlike ORFs.

A rough estimate of the odds in the link conservation can be expressed as the ratio of the two conservation ratios

$$C_F = c_F/c_D. \tag{27}$$

Its value is 1.62, that is the links between alike ORFs are 62% more likely to be conserved than the links between unlike ORFs ($c_F = 0.15$, $c_D = 0.09$). The p-value evaluated over the same graph with a randomised annotation list is 0.47.

More refined estimate of the odds which takes in consideration also the link statistics of the two networks uses mutual information, I_F and I_D. The mutual information expresses the level of correlation of the presence of the link in the two networks. If we normalise the frequencies n_F, $p_F(a,b) = n_F(a,b)/\sum_{c,d} n_F(c,d)$, we may write the mutual information as

$$I_F = \sum_{a,b} p_F(a,b) \ln \frac{p_F(a,b)}{p_F^A(a) p_F^B(b)}, \tag{28}$$

where the marginals are defined as $p_F^A(a) = \sum_b p_F(a,b)$ and $p_F^B(a) = \sum_a p_F(a,b)$. In the same way we define the mutual information I_D for the unlike ORFs pairs. For the subgraph of the sequence homologs the mutual information reads $I_F = 0.049$, $I_D = 0.005$. Defining the final information odds, $D_F = I_F/I_D$, we get $D_F = 9.58$ with the p-value 0.13 (the same test as for C_F). When we express these correlations in terms of Pearson correlations R_F and R_D we obtain $R_F = 0.25$ and $R_D = 0.13$.

References

[1] Uetz P, Dong Y-A, Zeretzke C, Atzler C, Baiker A, Berger B, Rajagopala SV, Roupelieva M, Rose D, Fossum E, Haas J (2006) Herpesviral protein networks and their interaction with the human proteome. *Science* **311**: 239–242.

[2] Berg J and Lässig M (2006), Cross-species analysis of biological networks by Bayesian alignment. *Proc Natl Acad Sci USA* **103(29)**: 10967–10972.

[3] Kolář M, Lässig M, and Berg J, (2008) On the statistical significance of graph alignments. *submitted.*

[4] Hiscock D, Upton C (2000) Viral Genome Database: A tool for storing and analyzing genes and proteins from complete viral genomes. *Bioinformatics* **16**: 484–485.

[5] Bao Y, Federhen S, Leipe D, Pham V, Resenchuk S, Rozanov M, Tatusov R, and Tatusova T (2004) National Center for Biotechnology Information Viral Genomes Project. *J Virol.* **78(14)**: 7291–7298.

[6] Mar Albà M, Lee D, Pearl FMG, Shepherd AJ, Martin N, Orengo CA, and Kellam P (2001) VIDA: a virus database system for the organisation of virus genome open reading frames. *Nuleic Acids Research* **29(1)**: 133–136.

[7] Needleman SB, Wunsch CD (1970) A general method applicable to the search for similarities in the amino acid sequence of two proteins *J. Mol. Biol.*, **48**, 443-453.

[8] Henikoff S and Henikoff JG (1992) Amino acid substitution matrices from protein blocks *Proc. Natl. Acad. Sci. USA*, **89**, 10915-10919.

[9] Yu Y-K and Hwa T, (2001) Statistical significance of probabilistic sequence alignment and related local Hidden Markov Models *Journal of Computational Biology*, **Vol. 8, Num. 3**, 249-282.

[10] Thompson JD, Higgins DG and Gibson TJ (1994) CLUSTAL W: improving the sensitivity of progressive multiple sequence alignment through sequence weighting, positions-specific gap penalties and weight matrix choice. *Nucleic Acids Research* **22**: 4673–4680.

[11] Vlček Č, Beneš V, Lu Z, Kutish GF, Pačes V, Rock D, Letchworth GJ and Schwyzer M, (1995) Nucleotide sequence analysis of a 30-kb region of the bovine herpesvirus 1 genome which exhibits a colinear gene arrangement with the UL21 to UL4 genes of herpes simplex virus *Virology* **210 (1)**: 100–108.

[12] Morgenstern B (2004) DIALIGN: Multiple DNA and protein sequence alignment at BiBiServ. *Nucleic Acids Research* **32**: W33–W36.

[13] Gotoh O (1996) Significant improvement in accuracy of multiple protein sequence alignments by iterative refinement as assessed by reference to structural alignments. *J. Mol. Biol.* **264**: 823–838.

[14] Edgar RC (2004) MUSCLE: multiple sequence alignment with high accuracy and high throughput. *Nucleic Acids Research* **32(5)**: 1792–97.

[15] Notredame C, Higgins D, Heringa J (2000) T-Coffee: A novel method for multiple sequence alignments. *Journal of Molecular Biology* **302**: 205–217.

[16] Sze S-H, Lu Y, and Yang Q (2006) A polynomial time solvable formulation of multiple sequence alignment. *Journal of Computational Biology* **13**: 309–319.

[17] Karplus K, Barrett C, and Hughey R (1998) Hidden Markov Models for detecting remote protein homologies. *Bioinformatics* **14(10)**: 846–856.

[18] Lipman D, Altschul S, and Kececioglu J (1989) A Tool for Multiple Sequence Alignment *Proc. Natl. Acad. Sci. USA* **86**: 4412–4415.

[19] Calderwood MA, Venkatesan K, Xing L, Chase MR, Vazquez A, Holthaus AM, Ewence AE, Li N, Hirozane-Kishikawa T, Hill DE, Vidal M, Kieff E, and Johannsen E (2007) Epstein–Barr virus and virus human protein interaction maps *Proc. Natl. Acad. Sci. USA* **104**: 7606–7611.
